# Growth, yield and nutrient-use responses of drought-tolerant maize varieties to sowing date and fertilizer microdosing in northern Benin

**DOI:** 10.1371/journal.pone.0361710

**Published:** 2026-10-28

**Authors:** Mouiz W. I. A. Yessoufou, Pierre G. Tovihoudji, Sissou Zakari, P. B. Irénikatché Akponikpè

**Affiliations:** 1 Laboratory of Hydraulics and Environmental Modeling (HydroModE Lab), Faculty of Agronomy (UP/FA), Université de Parakou, Parakou, Bénin; 2 Institute of Science and Technology for Innovation in Africa (ISTI-Africa), Parakou, Benin; University of Minnesota, UNITED STATES OF AMERICA

## Abstract

Changing sowing dates has been a farming low-cost strategy to mitigate climate induced water stress during the juvenile stage of crops. This study evaluates the potential of drought-tolerant maize varieties and fertilizer microdosing to enhance maize productivity under different sowing dates in rainfed conditions. We conducted a two-year strip split-plot trial with three sowing dates (early, normal, and late), five maize varieties (DMR, TZEE, TZE, 2008Syn, and 2000Syn) and five fertilizer treatments (control: no fertilizer, three microdosed rates MD1: 25 kg N + 4 kg P ha^-1^, MD2: 25 kg N + 8 kg P ha^-1^, MD3: 35 kg N + 8 kg P ha^-1^, and the recommended rate RR: 76 kg N + 13 kg P ha^-1^). DMR is a drought-susceptible variety; and TZEE, TZE, 2008Syn, and 2000Syn are drought-tolerant. Results show that grain yield was 9.9% higher and 10.2% lower under early and late sowing, respectively, compared to normal sowing. Grain yield was 29.9, 37.8, 26.5 and 12.5% higher under TZEE, TZE, 2008Syn, and 2000Syn, respectively compared to DMR; and 153.3, 200.9, 247.2 and 220.8% under MD1, MD2, MD3 and RR, respectively compared to control. DMR achieved its highest mean grain yield under the recommended fertilizer rate, whereas the drought-tolerant varieties achieved their highest mean yields under MD3. The findings confirm that late sowing reduced maize performance, while drought-tolerant varieties and fertilizer microdosing independently improved average productivity.

## Introduction

Maize (*Zea mays L.*) is a major staple crop contributing significantly to food security and rural livelihood in sub-Saharan Africa (SSA), (50% of cereal production and 30% of daily calorie intake) [1,2]. However, its production faces substantial yield variability caused by climate variability and change, such as extreme rainfall patterns and dry spells [3,4]. Such climatic shifts highly affect the dynamics of maize growth and yield [5], leading to serious threat to agricultural sustainability and food security in Benin. It is therefore necessary to identify and validate effective climate-smart management practices that can sustain maize productivity under changing climatic conditions. Adaptation strategies to address climate-induced yield variability include variety management (drought-tolerant cultivars), soil and nutrient management (conservation agriculture, agroforestry, organic manure application, mineral fertilization, integrated soil fertility management), and sowing date optimization [6–8]. Among all these strategies, the use of drought-tolerant varieties, mineral fertilization and sowing date adjustment has been particularly promoted in SSA [8–10].

Adjusting sowing dates is a low-cost strategy that aligns crop growth with periods of adequate rainfall, thereby reducing the risk of exposure to adverse climatic conditions during sensitive developmental stages [11,12]. Many farmers in the SSA region already adopt flexible sowing dates as an adaptive response to climate variability [13]. However, its effective implementation depends on timely and reliable climate information [13], which remains inaccessible to many smallholder farmers in West Africa [14]. Late sowing may cause the crop to exhaust available soil moisture before completing critical phenological stages [15], and may increase vulnerability to pest infestations and disease outbreaks when vegetative growth coincides with peak pest pressure [16]. Despite these risks, delayed sowing remains frequent because of uncertain rainfall onset and peak-season labour constraints that prevent timely crop establishment [17]. Under such conditions, identifying management practices that sustain crop performance when sowing is delayed becomes vital.

Drought-tolerant maize varieties are bred to maintain stable grain production when rainfall is insufficient or poorly distributed [18]. Their advantage results from traits such as improved root development, delayed leaf senescence, sustained photosynthesis, better water-use efficiency, and more stable assimilate partitioning under stress [19,20]. Recent evidence shows that stress-tolerant germplasm has contributed to maize yield gains and risk reduction across sub-Saharan Africa, with reported yield advantages of about 15–33% in several countries [21]. However, varietal response remains dependent on environmental conditions and crop management [22]. It is therefore important to evalute the response of multiple drought-tolerant genotypes across contrasting management strategies.

Mineral fertilizers application improves crop growth and yield in the nutrient-depleted soils by providing readily available nutrients to plants [23,24]. However, yield and efficiency do not respond proportionally to increasing fertilizer rates [25]. Higher fertilizer rates may maximize biomass or grain yield but often reduce nutrient use efficiency [26]. Fertilizer microdosing consists of placing a small quantity of mineral fertilizer in or near each planting hole at sowing or shortly after crop emergence [27,28]. Unlike simply reducing the fertilizer rate uniformly across a field, it combines a reduced dose with localized placement near the root zone to improve fertilizer-use efficiency and reduce input requirements [27,29]. In fact, Kouadio et al. [30] reported a 73–112% yield gain following DAP and NPK microdosing on cereals in Niger. Despite these benefits, widespread adoption remains constrained by the high labour requirements of precise fertilizer placement and farmers’ limited access to technical and climate information [28]. To improve its effectiveness, microdosing has been promoted in combination with drought-tolerant maize. Tovihoudji et al. [8] demonstrated that integrating drought-tolerant varieties with fertilizer microdosing significantly improved maize growth, grain and stover yields, and gross economic returns in Benin.

Despite the evidence supporting drought-tolerant varieties and fertilizer microdosing, empirical evidence of their combined effectiveness under adjusted sowing conditions is still limited. Most studies in sub-Saharan Africa have evaluated these practices individually, providing limited evidence on their combined effects. Hence, it remains unclear whether drought-tolerant varieties and fertilizer microdosing can mitigate the reductions in maize productivity associated with delayed sowing. Consequently, the extent to which the benefits of drought-tolerant varieties and fertilizer microdosing are additive or dependent on sowing conditions remains insufficiently understood, particularly under the agroclimatic conditions of northern Benin. Addressing this gap is essential for developing evidence-based strategies to enhance maize resilience, productivity, and food security under changing climatic conditions.

The objective of this study was to determine whether drought-tolerant varieties and fertilizer microdosing can sustain maize productivity and nutrient-use efficiency under variable sowing dates in northern Benin. We hypothesized that: (i) delayed sowing would reduce maize growth, yield and nutrient-use efficiency; (ii) drought-tolerant varieties would perform better than the drought-susceptible variety under delayed sowing; (iii) fertilizer microdosing would increase yield and nutrient-use efficiency compared to the unfertilized control; and (iv) combining drought-tolerant varieties with fertilizer microdosing would mitigate the yield penalty associated with delayed sowing. The findings will provide insights for optimizing agronomic practices to strengthen maize-based production systems in the context of climate change.

## Materials and methods

### Experimental site

A two-year experimentation (2018–2019) was conducted in Northern Benin at the Agricultural Research Center in Ina village (9°57’N and 2°42’E, 365 m a.s.l). The study area belongs to the Sudanese climate characterized by one rainy season extending from May to October and a dry season from November to April. Annual rainfall ranges from 900 to 1,300 mm, while the mean daily temperature is approximately 27.5°C. The soil is tropical ferruginous and classified as sandy loam, with mineral-deficiency and low fertility (Table 1). The soil is slightly acidic, with low organic matter, total nitrogen, available phosphorus, and exchangeable potassium (Table 1).

**Table 1:** Physicochemical characteristics of the topsoil (0-20??cm) at the experimental site.

	Characteristics	Value
**Physical**	Clay (%)	4.00
	Silt (%)	14.00
	Sand (%)	82.00
	Bulk density (g/cm³)	1.50
**Chemical**	pH-H_2_O	5.70
	Organic carbon (%)	0.41
	Total nitrogen (mg/kg)	397.10
	C/N ratio	10.30
	Available P (mg/kg)	11.60
	Exchangeable K (cmol/kg)	0.21

### Experimental setup

The experimental design was a strip-split-plot with three replicates arranged as blocks. Sowing date was the strip factor (main plots) and comprised three levels: early sowing, normal sowing and late sowing. Fertilizer was the split-plot factor (subplots) and comprised five treatments: an unfertilized control; microdose rate 1 (MD1: 25 kg N plus 4 kg P ha^−1^); microdose rate 2 (MD2: 25 kg N plus 8 kg P ha^−1^); microdose rate 3 (MD3: 35 kg N plus 8 kg P ha^−1^); and the recommended rate by the National Agricultural Research Institute of Benin INRAB (RR: 76 kg N plus 13 kg P ha^−1^). Variety was the sub-subplot factor and comprised one drought-sensitive variety, DMR-ESR-W, and four drought-tolerant varieties: TZEE-W POP STR QPM (TZEE), TZE-Y POP STR (TZE), 2008 Syn EE-W DT STR (2008Syn) and 2000Syn EE-W (2000Syn). Within each replicate, one sowing-date plot (29 m wide × 33 m long) was established for each of the three sowing dates. The three sowing-date treatments were randomly assigned to the main plots. Each sowing-date plot was divided into five randomly assigned fertilizer plots measuring 29 m wide × 5 m long with 2 m alleys. Each fertilizer plot was further divided into five variety sub-subplots measuring 4 m wide × 5 m long with 2 m alleys. The five maize varieties were randomly allocated to the sub-subplots within each fertilizer plot.. The variety subplot was the smallest experimental unit used for crop measurements and statistical analysis, while individual plants sampled within each subplot were considered subsamples. The factorial combination of the sowing dates, fertilizer treatments and maize varieties resulted in 75 treatment combinations and 225 elementary plots.

### Crop management and fertilizer application

The experiment was conducted entirely under rainfed conditions. In the season preceding trial establishment, the field was cultivated with maize without fertilizer application. Field preparation was identical for all treatments and seasons. The soil was tilled using a 20 cm tractor-mounted disk plough before planting. Plots were manually leveled with rakes at sowing. Planting occurred on the 6^th^ June (early sowing), 3^rd^ July (normal sowing) and 21^st^ July (late sowing) in 2018 and on the 9^th^ June (early sowing), 6^th^ July (normal sowing) and 24^th^ July (late sowing) in 2019 (Fig 1). Three to four seeds were sown in hills arranged at 0.8 m between rows and 0.4 m within rows, giving six rows per subplot and 13 planting pockets per row. Seedlings were thinned to two plants per hill at two weeks after emergence, to reach a recommended density of 62,500 plants ha^-1^.

**Fig 1:**
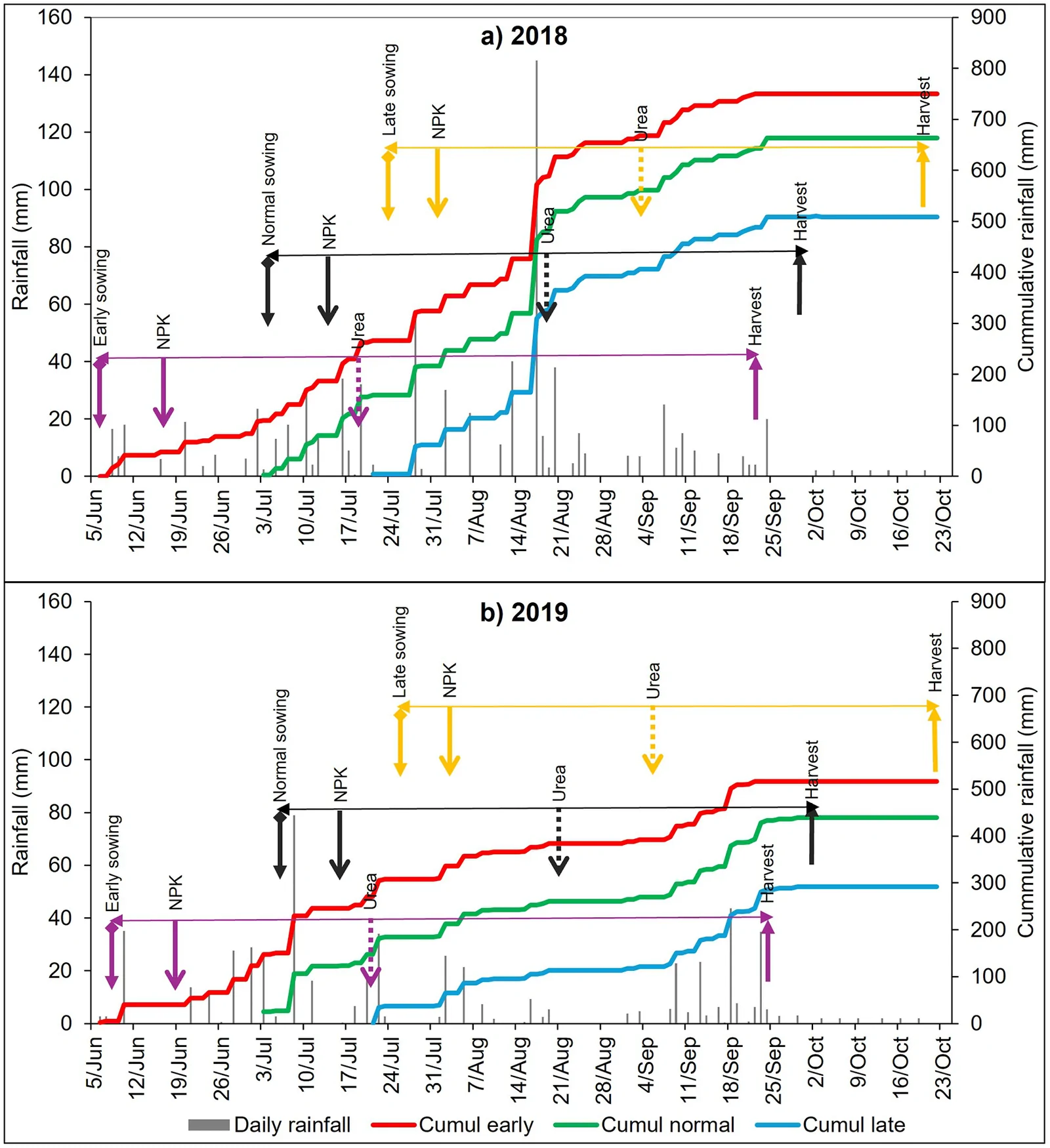
Rainfall pattern and main crop management events for each sowing dates during the growing season of a) 2018 and b) 2019.

Potassium was applied uniformly to all plots at 25 kg ha^-1^. Phosphorus and potassium were applied once at 10 days after sowing (DAS), using triple superphosphate (46% P_2_O_5_) and muriate of potash (60% K_2_O). Nitrogen was supplied as urea, with half applied at 10 DAS and the remainder at 45 DAS. Fertilizer was broadcast at 10 cm from each planting hill for the recommended rate treatment and incorporated at 5 cm deep and 5 cm away from each planting hill for the microdose treatments, according to Tovihoudji et al. [31]. Specifically, MD1 corresponded to 1.7 g urea, 0.6 g triple superphosphate and 1.6 g muriate of potash per hill; MD2 to 1.7, 1.3 and 1.6 g hill^-1^, respectively; and MD3 to 2.4, 1.3 and 1.6 g hill^-1^, respectively.

The plots were weeded manually at 15 and 30 DAS. Ridging was performed at 45 DAS using a hand hoe after the second urea application. Maize was harvested on the 22^nd^ September (early sowing), 1^st^ October (normal sowing) and 19^th^ October (late sowing) in 2018 and on the 25^th^ September (early sowing), 4^th^ October (normal sowing) and 22^th^ October (late sowing) in 2019 (Fig 1).

### Data collection

#### Soil analysis.

Prior to the establishment of the trials, composite soil samples were collected from the 0–20 cm horizon across the study site. Soil was extracted from 10 randomly selected locations using a zig-zag pattern. The collected soil was thoroughly mixed to form composite samples, which were then air-dried and sieved through a 2 mm mesh. The soil samples were tested for particle size, pH in water (pH-H_2_O), organic carbon content, total nitrogen, available phosphorus, and exchangeable potassium. Particle size distribution was determined according to Gee and Or [32]. The total nitrogen was determined according to Houba et al. [33]. The soil pH, available phosphorus and exchangeable potassium were determined according to van Reeuwijk [34]. The organic carbon was quantified following the Walkley and Black method [35], and organic matter content was estimated based on the organic carbon content using the Van Bemmelen factor as described by Ezzaouini et al. [36]. All soil chemical analyses were conducted at the Soil and Plant Analysis Laboratory of the National Institute for Agricultural Research of Benin (INRAB) of the Agonkanmè Regional Agricultural Research Center (CRA) in Benin.

#### Maize growth and yield.

Plant growth was evaluated using plant height, number of leaves, leaf area, and aboveground total dry matter (TDM). Measurements were taken biweekly starting from 20 days after sowing (DAS) on randomly selected plants within an 8.64 m² sampling area in each elementary plot.

Plant height was measured on five plants from the base to the last visible ligule using a meter ruler, up to the flowering stage. Leaf area and leaf area index (LAI) were determined on three plants within the sampling area by measuring leaf length and maximum width, following the method of Wang et al. [37]:


$$\begin{array}{c} \text{Leaf area}=\text{L}\times \text{W}\times \text{k} \end{array}$$ (1)

where L is the leaf length, W is the maximum width, and k is a shape factor with the value of 0.75. The LAI was subsequently calculated as the ratio of leaf area to the horizontal soil surface area occupied by each planting hill [38].

The aboveground total dry matter of two whole plants was determined by the destructive method; samples were initially oven-dried at 105 °C for 30 minutes, followed by further drying at 70 °C until a constant weight was achieved, expressed in g m^-2^. Grain and biomass yield measurements were done at plant maturity. Harvesting was done manually within the harvest area of each elementary plot. The number of plants, the total number of ears, and the number of filled and unfilled cobs, the total weight of the cobs and the grain moisture content were measured and recorded. The grain, stover and biomass yields were then estimated as described by Tovihoudji et al. [8].

#### Nutrient productivities.

The agronomic efficiency (AE) and partial factor productivity (PFP) of nitrogen (N) and phosphorus (P) were calculated to assess nutrient use productivity in maize. Both indices were computed following the formulas described by Rawal et al. [39]:


$$\begin{array}{c} {AE}_{i}=\frac{\left(Yt\;-\;Yc\right)}{Qt} \end{array}$$ (2)

$$\begin{array}{c} {PFP}_{i}=\frac{Yt}{Qt} \end{array}$$ (3)

where *Yt* and *Yc* represent the yield for the treatment and control, respectively. Qt represents the quantity of nutrients *i* (N or P) applied.

#### Rainfall data.

Daily rainfall was measured using a rain gauge positioned at the experimental field throughout the two-year study period. A dry day was defined as a day receiving less than 1 mm of rainfall [40,41], and a dry spell was defined as three or more consecutive dry days [42,43].

### Data analysis

The statistical analyses were performed with the R statistical software (version 4.5.2). For each response variable, a combined linear mixed-effects model was fitted using the *lmer* function of the *lmerTest* package [44]. Year, sowing date, fertilizer, variety and all their interactions were included as fixed effects. Year was treated as a fixed effect because the experiment comprised only two specific growing seasons (2018 and 2019), and the objective was to explicitly test differences between these seasons and their interactions with the experimental treatments rather than to estimate variance among a broader population of years. The model was specified as:


$$\begin{array}{cc} esponse\;\sim \;Year & \times Sowing\;date\;\times \;Fertilizer\;\times \;Variety+(\text{1}|Year:Block\\ & +(\text{1}|Year:Block:Sowing\;date)+(\text{1}|Year:Block:Sowing\;date:Fertilizer) \end{array}$$

Sowing date within block and year represented the main-plot error, while fertilizer within sowing date, block and year represented the subplot error. The residual variance represented the sub-subplot error used for testing variety effects and interactions involving variety. Type III F-tests were performed using the Kenward-Roger approximation for denominator degrees of freedom. Model assumptions were assessed before analysis. Residual normality was evaluated using the Anderson-Darling test with the *ad.test* function of the *nortest* package [45], while homogeneity of variance was assessed using Levene’s test with the *leveneTest* function of the *car* package [46]. Total dry matter was square-root transformed ($\sqrt{x}$), grain yield was square transformed ($x^{\text{2}}$), and phosphorus agronomic efficiency and partial factor productivity were log-transformed [log(x)]. Statistical tests were conducted on the transformed scale, while estimated marginal means and confidence intervals were presented after back-transformation to the original measurement scale. Where significant effects were detected, estimated marginal means were calculated using the *emmeans* package and compared using Tukey-adjusted pairwise comparisons at the 5% significance level. For the growth variables, the analysis of the interaction between sowing date, maize variety, and fertilizer levels specifically focused on the anthesis stage (62 DAS). To understand the response of maize growth, yield and nutrient productivities under varying sowing date, drought-tolerant varieties and fertilization, a redundancy analysis (RDA) was performed for each variety, using the *rda* function in the *vegan* package in R [47].

## Results

### Rainfall characteristics

The total rainfall was 678.4 mm, 573.2 mm, and 418.1 mm for early, normal and late sowing during the 2018; and 516.3 mm, 439.3 mm, and 291.7 mm for early normal late sowing during the 2019 growing season, respectively. In terms of frequency, 41, 33, and 24 rainfall events were recorded under early, normal and late sowings in 2018; and 34, 31, and 25 rainfall events in 2019 respectively (Fig 1 and Table 2). Maize crops received the highest rainfall amount during the silking stage for early and normal sowing, and during the active vegetative phase for late sowing in 2018 (Table 2). However, the highest rainfall mainly happened during the active growth stage under early sowing, juvenile stage under normal sowing, and milking stage under late sowing in 2019. Dry spells were 33% and 40% more frequent under early sowing (12 in 2018 and 7 and 2019) and 9% and 17% under late sowing (11 in 2018 and 6 in 2019) compared to normal sowing (9 in 2018 and 5 in 2019), respectively; indicating variable moisture stress conditions across sowing periods and years (Table 2).

**Table 2:** Rainfall and dry spells characteristics during the growing season of 2018 and 2019.

		2018			2019		
	Phenology	Early	Normal	Late	Early	Normal	Late
Cumulative rain (mm)	Juvenile	77.5	159.1	86.5	66.5	184.3	86.3
	Active growth stage	92.15	82.5	174	162.9	49.6	21.5
	Tasseling stage	60.6	15	60.5	23.2	9.2	5.4
	Silking	118.5	241.6	63.1	104.3	26.1	36.9
	Milking stage	215	58	28	26.9	65.8	89.4
	Maturity	114.6	17	6	132.5	104.3	52.2
	Total season	678.4	573.2	418.1	516.3	439.3	291.7
Rain events (number and range(mm))	Juvenile	7 (4 - 19)	10 (1 - 34)	5 (3 - 55)	5 (1 - 35)	8 (1 - 79)	5 (3 - 34)
	Active growth stage	6 (2 - 29)	4 (3 - 55)	4 (4 - 145)	5 (3 - 79)	3 (3 - 26)	4 (1 - 9)
	Tasseling stage	4 (1 - 34)	2 (4 - 11)	4 (3 - 38)	2 (1 - 16)	2 (2 - 7)	1 (5 − 5)
	Silking	6 (3 - 55)	9 (3 - 145)	5 (1 - 25)	6 (3 - 34)	5 (1 - 10)	4 (4 - 23)
	Milking stage	6 (3 - 145)	4 (1 - 25)	4 (4 - 9)	5 (1 - 9)	6 (1 - 23)	6 (1 - 44)
	Maturity	12 (1 - 25)	4 (2 - 7)	2 (2 - 4)	11 (1 - 44)	7 (1 - 44)	5 (3 - 35)
	Total season	41 events	33 events	24 events	34 events	31 events	25 events
Dry spells events (3 days or more)	Juvenile	1	1	3	1	1	1
	Active growth stage	1	3	2	1	1	1
	Tasseling stage	1	1	0	1	1	1
	Silking	3	1	3	1	1	2
	Milking stage	2	2	2	1	1	0
	Maturity	4	1	1	2	0	1
	Total season	12	9	11	7	5	6

### Effect of sowing date, drought tolerant varieties and fertilizer microdosing on maize growth

Plant height increased progressively with DAS across all treatments, with the most rapid growth occurring between 34 and 48 DAS, followed by a slower rate of increase toward anthesis at 62 DAS (Fig 2). Plant height was significantly influenced by sowing date at all measurement intervals (*P* < 0.05; Fig 2). At 62 DAS, plant height increased by 10% under early and 4% under late sowing compared to normal sowing (Fig 2). Significant differences among varieties were also observed at all DAS, except for 20 DAS (*P* < 0.05; Fig 3). Drought-tolerant maize had taller plants except for 2008Syn compared to DMR, at 62 DAS. Fertilizer application had no significant effect on plant height at 20 and 34 DAS but became significant at 48 and 62 DAS (*P* < 0.05). At anthesis, plant height was 13, 9, 18 and 13% higher under MD1, MD2, MD3 and RR compared to the unfertilized control, respectively (Fig 2). Plant height at 62 DAS differed with sowing date by fertilizer interaction (*P* < 0.001; Fig 3a). Indeed, the increase in plant height due to fertilizer was higher under early sowing (44% compared to unfertilized control) than normal and late sowing (10 and 7%, respectively). Furthermore, plant height at 62 DAS differed with variety by fertilizer interaction (*P* < 0.001; Fig 3b). The plant height was 25, 23 and 22% higher under MD3 than unfertilized control for DMR, TZE and 2000Syn respectively, but there was no significant difference in plant height for all fertilizer treatments under TZEE and 2008Syn.

**Fig 2:**
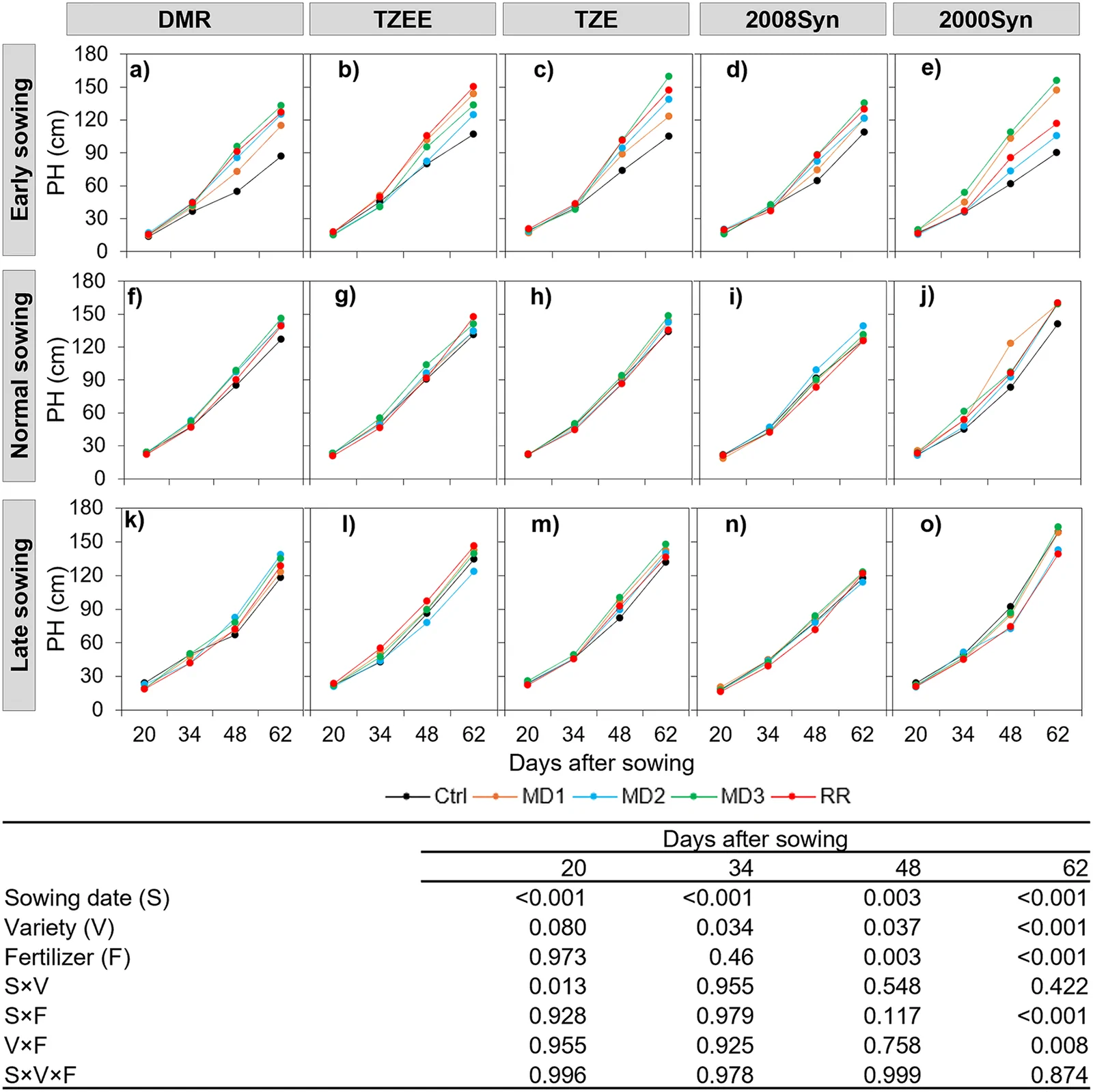
Evolution of plant height for five maize varieties at varying sowing dates and fertilizer applications. PH: plant height. Ctrl: unfertilized, MD1: 25 kg N + 4 kg P ha^-1^, MD2: 25 kg N + 8 kg P ha^-1^, MD3: 35 kg N + 8 kg P ha^-1^, RR: 76 kg N + 13 kg P ha^-1^. The numbers in the table represent the p value.

**Fig 3:**
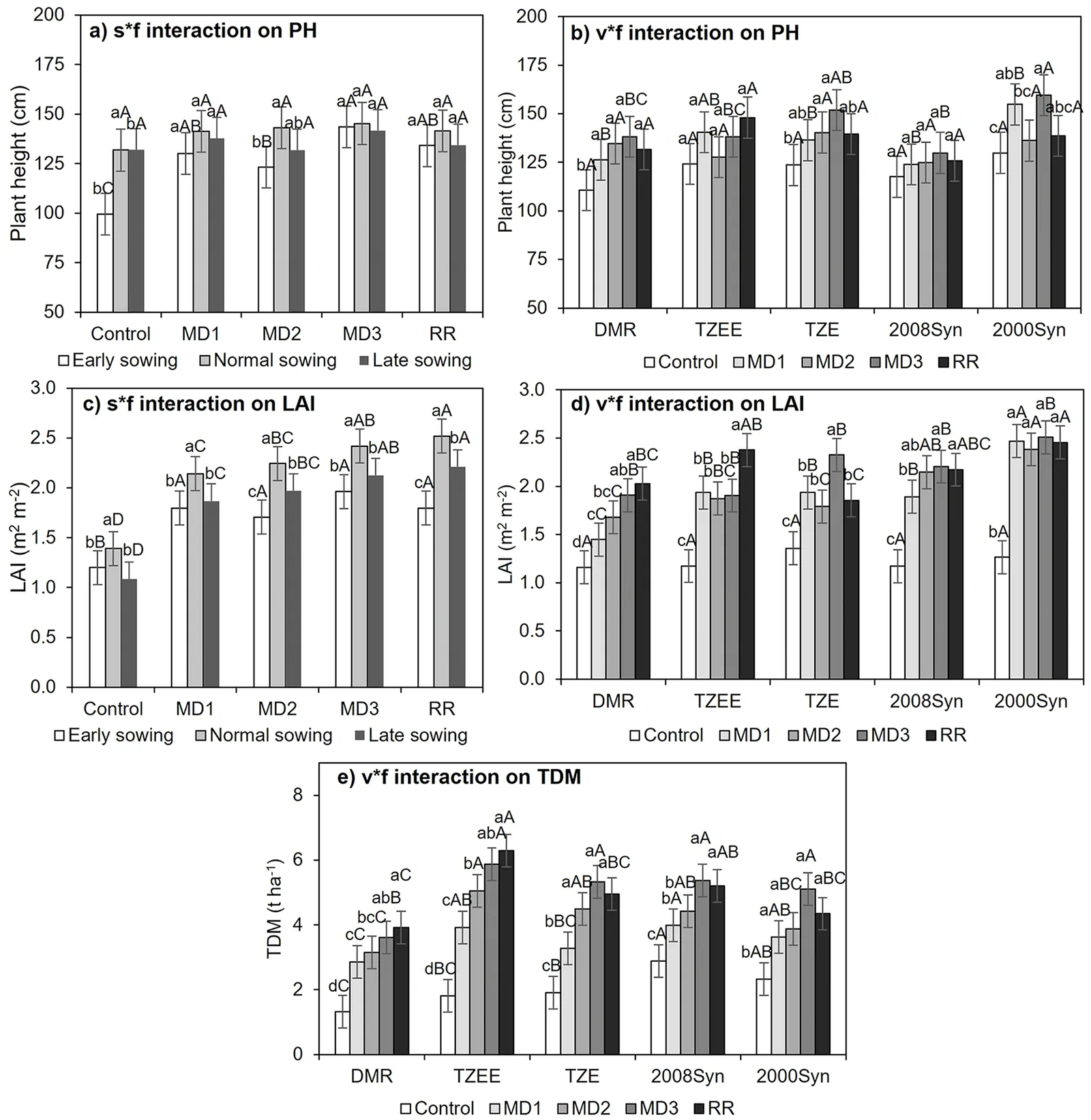
Plant height (a, b), Leaf area index (c, d), and Total dry matter (e) as affected by the interaction between sowing date, fertilizer and maize variety. PH: plant height, LAI: leaf area index, TDM: total dry matter. Control: unfertilized, MD1: 25 kg N + 4 kg P ha^-1^, MD2: 25 kg N + 8 kg P ha^-1^, MD3: 35 kg N + 8 kg P ha^-1^, RR: 76 kg N + 13 kg P ha^-1^. Error bars represent the standard error. Letters “abc” indicate mean separation among the grouped bars, while letters “ABC” indicates mean separation among factor represented in the legend.

The leaf area index increased with DAS, peaked between 62 and 76 DAS, and declined thereafter towards maturity at 90 DAS (Fig 4). LAI was significantly affected by sowing date at all measurement dates (*P* < 0.001; Fig 4). The maximum LAI (at 62 DAS) decreased by 24% under early sowing and 14% under late sowing, compared to normal sowing (Table 3). LAI was also significantly influenced by variety at all DAS, except for 20 DAS (*P* < 0.001; Fig 4). Drought-tolerant varieties generally had higher LAI than DMR at 62 DAS. Fertilizer application also significantly affected LAI at all stages (*P* < 0.001; Fig 3). At anthesis (62 DAS), LAI was 58% higher under MD1 and MD2 than unfertilized control (Table 3); and by 83% for MD3 and RR compared to unfertilized control (Fig 4). The LAI at 62 DAS differed significantly with sowing date by fertilizer interaction (*P* < 0.001; Fig 3c). The highest LAI resulting from fertilizer application was observed in MD3 for early sowing (1.9 cm^2^ cm^-2^) and in RR for normal (2.5) and late sowing (2.2). Similarly, LAI at 62 DAS varied with variety by fertilizer interaction (*P* = 0.021; Fig 5d). The highest LAI under DMR (2.0) and TZEE (2.4) was recorded under RR treatment, while LAI was highest for TZE (2.3), 2008Syn (2.2) and 2000Syn (2.5) under MD3 fertilizer.

**Table 3:** Summary of the main effects of year, sowing date, variety and fertilizer on yield and efficiency over the two cropping years.

	Grain yield (t ha^-1^)	Stover yield (t ha^-1^)	Agronomic efficiency of N (kg kg^-1^)	Agronomic efficiency of P (kg kg^-1^)	Partial factor productivity of N (kg kg^-1^)	Partial factor productivity of P (kg kg^-1^)
**Year (Y)**						
2018	2.3a	4.2a	50.85a	243.36a	78.18a	363.93a
2019	2.1b	4.1a	51.35a	236.99a	75.56a	351.71a
**Sowing date (S)**						
Early	2.4a	4.5a	53.69a	248.59a	83.64a	390.54a
Normal	2.2b	4.2b	48.89b	225.97b	76.39b	356.28b
Late	2.0c	3.8c	50.7ab	232.5aa	70.58c	326.67c
**Variety (V)**						
DMR	1.8d	3.4d	44.28c	202.48b	62.17d	287.24d
TZEE	2.4b	4.2cb	54.49ab	254.63a	82.75b	388.56ab
TZE	2.5a	4.9a	60.05a	278.17a	88.07a	410.95a
2008Syn	2.3b	4.4b	49.44cb	225.72b	79.97b	370.41b
2000Syn	2.1c	4.0c	47.22c	217.44b	71.39c	331.99c
**Fertilizer (F)**						
Control	0.8e	2.3d	–	–	–	–
MD1	2.2d	3.9c	51.94c	324.59a	85.81b	536.31a
MD2	2.5c	4.7b	68.05a	212.67c	101.93a	318.53c
MD3	2.9a	5.1a	59.8b	261.64b	83.99b	367.49b
RR	2.7b	4.9a	24.61d	143.85d	35.75c	208.99d
***P-values***						
Y	***<0.001***	*0.767*	*0.720*	*0.707*	*0.086*	*0.063*
S	***<0.001***	***<0.001***	***0.009***	***0.013***	***<0.001***	***<0.001***
V	***<0.001***	***<0.001***	***<0.001***	***<0.001***	***<0.001***	***<0.001***
F	***<0.001***	***<0.001***	***<0.001***	***<0.001***	***<0.001***	***<0.001***
Y × S	***0.040***	*0.620*	*0.242*	*0.228*	*0.077*	*0.117*
Y × V	*0.799*	*0.589*	*0.783*	*0.820*	*0.764*	*0.826*
Y × F	*0.384*	*0.549*	*0.569*	*0.465*	*0.831*	*0.865*
S × V	*0.296*	*0.569*	*0.972*	*0.984*	*0.989*	*0.994*
S × F	*0.059*	***0.005***	*0.775*	*0.723*	*0.432*	*0.071*
V × F	***<0.001***	***<0.001***	***0.007***	***<0.001***	***<0.001***	***<0.001***
Y × S × V	*0.852*	*0.892*	*0.834*	*0.863*	*0.824*	*0.884*
Y × S × F	*0.955*	*0.788*	*0.981*	*0.944*	*0.920*	*0.756*
Y × V × F	*0.991*	*0.998*	*0.999*	*0.993*	*0.999*	*0.999*
S × V × F	*0.999*	*0.999*	*0.999*	*0.999*	*0.999*	*0.999*
Y × S × V × F	*0.999*	*0.999*	*0.999*	*0.999*	*0.999*	*0.999*

Letters “abc” represents the mean separation amon factor levels (a > b > c).

**Fig 4:**
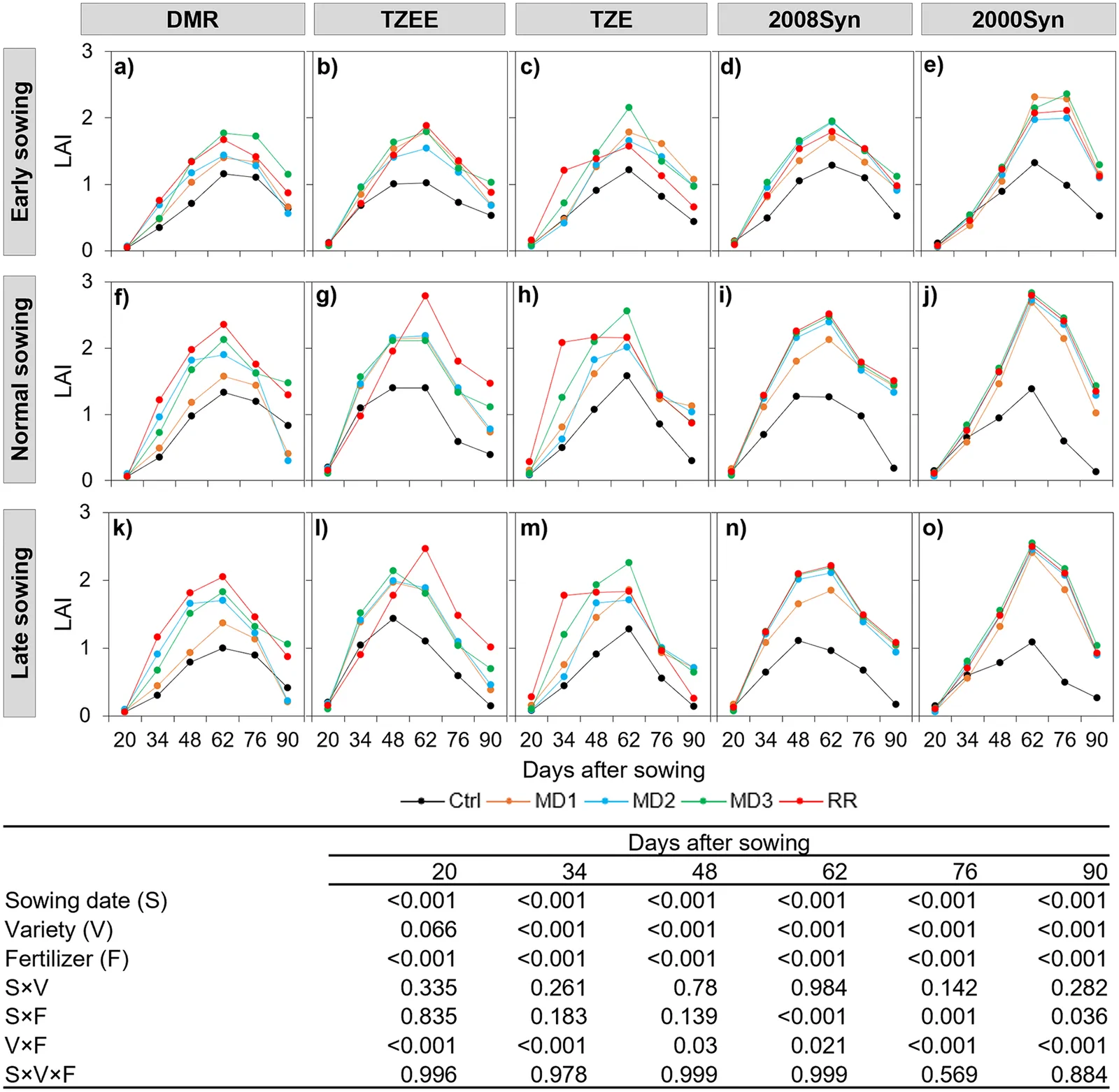
Evolution of leaf area index for five maize varieties at varying sowing dates and fertilizer applications. LAI: leaf area index. Ctrl: unfertilized, MD1: 25 kg N + 4 kg P ha^-1^, MD2: 25 kg N + 8 kg P ha^-1^, MD3: 35 kg N + 8 kg P ha^-1^, RR: 76 kg N + 13 kg P ha^-1^. The numbers in the table represent the *p* value.

**Fig 5:**
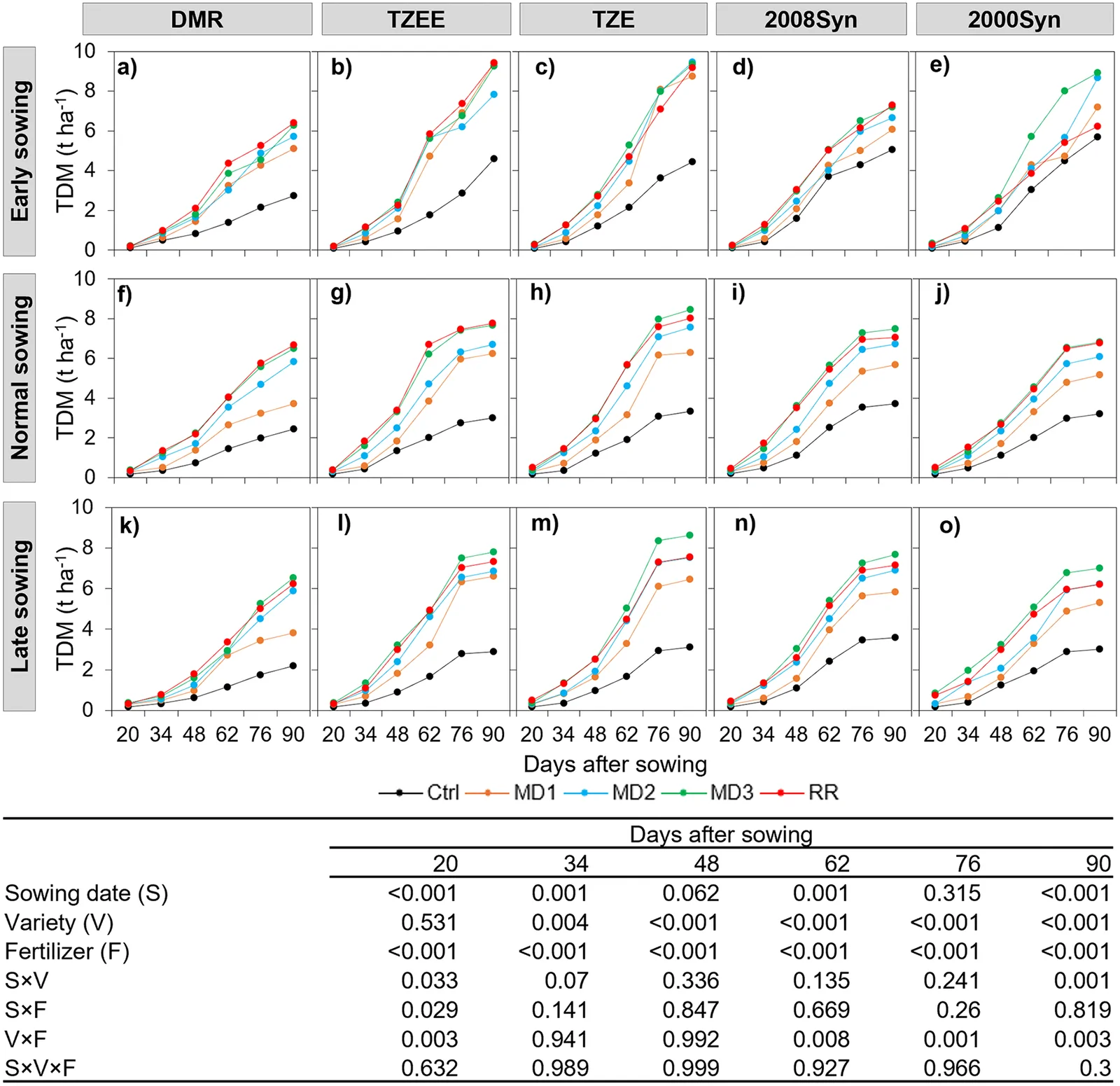
Evolution of total dry matter for five maize varieties at varying sowing dates and fertilizer applications. TDM: total dry matter. Ctrl: unfertilized, MD1: 25 kg N + 4 kg P ha^-1^, MD2: 25 kg N + 8 kg P ha^-1^, MD3: 35 kg N + 8 kg P ha^-1^, RR: 76 kg N + 13 kg P ha^-1^. The numbers in the table represent the p value.

The above ground total dry matter increased progressively with DAS across all treatments, with the most rapid accumulation occurring between 48 and 76 DAS (Fig 5). The TDM was significantly influenced by sowing dates at all stages (*P* < 0.001; Fig 5), except at 48 and 76 DAS. At 62 DAS, TDM was 9% lower under late sowing than normal sowing while there was no significant difference in TDM between early and normal sowing (Fig 4). Variety significantly affected TDM at all DAS, except for 20 DAS (*P* < 0.05; Fig 5). TDM increased in the range of 30−54% under drought-tolerant maize compared to DMR (Table 3). TDM was also significantly affected by fertilizer application at all stages (*P* < 0.001; Fig 5). TDM was 72, 105, 147 and 141% higher under MD1, MD2, MD3 and RR compared to unfertilized control, respectively at 62 DAS (Fig 5). The TDM at 62 DAS differed with variety by fertilizer interaction (*P* = 0.008; Fig 3e). Indeed, TDM was higher for DMR (3.9 t ha^-1^) and TZEE (6.3 t ha^-1^) under RR fertilizer, while TZE (5.3 t ha^-1^), 2008 Syn-EE (5.4 t ha^-1^) and 2000 Syn-EE (5.1 t ha^-1^) under MD3.

### Effect of sowing date, drought tolerant varieties and fertilizer microdosing on maize grain and stover yield

Grain yield was 8.7% lower in 2019 than in 2018 (*P* < 0.001; Table 3). Grain yield was 9.9% higher under early sowing and 10.2% lower under late sowing compared with normal sowing (*P* < 0.001; Table 3). GY was higher under all drought-tolerant varieties than DMR, with yield increases of 30%, 38%, 26%, and 12% for TZEE, TZE, 2008Syn, and 2000Syn, respectively (*P* < 0.001; Table 3). Across fertilizer treatments, grain yield followed the order MD3 > RR > MD2 > MD1 (P < 0.001; Table 3). There was a significant year × sowing date interaction (*P* < 0.001; Fig 6a). Grain yield was 9.5% lower in 2019 than in 2018 under early sowing, while there was no siginifacnt difference between grain of both years under normal and late sowing (Fig 6a). A significant variety × fertilizer interaction was observed (*P* < 0.001; Fig 6b), with the highest yield recorded under RR for DMR (2.6 t ha^-1^) and under MD3 for all drought-tolerant varieties (2.7–3.3 t ha-1). SY followed similar trends as GY (Table 3 and Fig 6c), except for the significant sowing date × fertilizer interaction (*P* = 0.005, Table 3). The highest SY under early sowing occurred with RR, while normal and late sowing achieved maximum SY (5.0 and 4.9 t ha^-1^, respectively) under MD3 fertilizer (Fig 6d).

**Fig 6:**
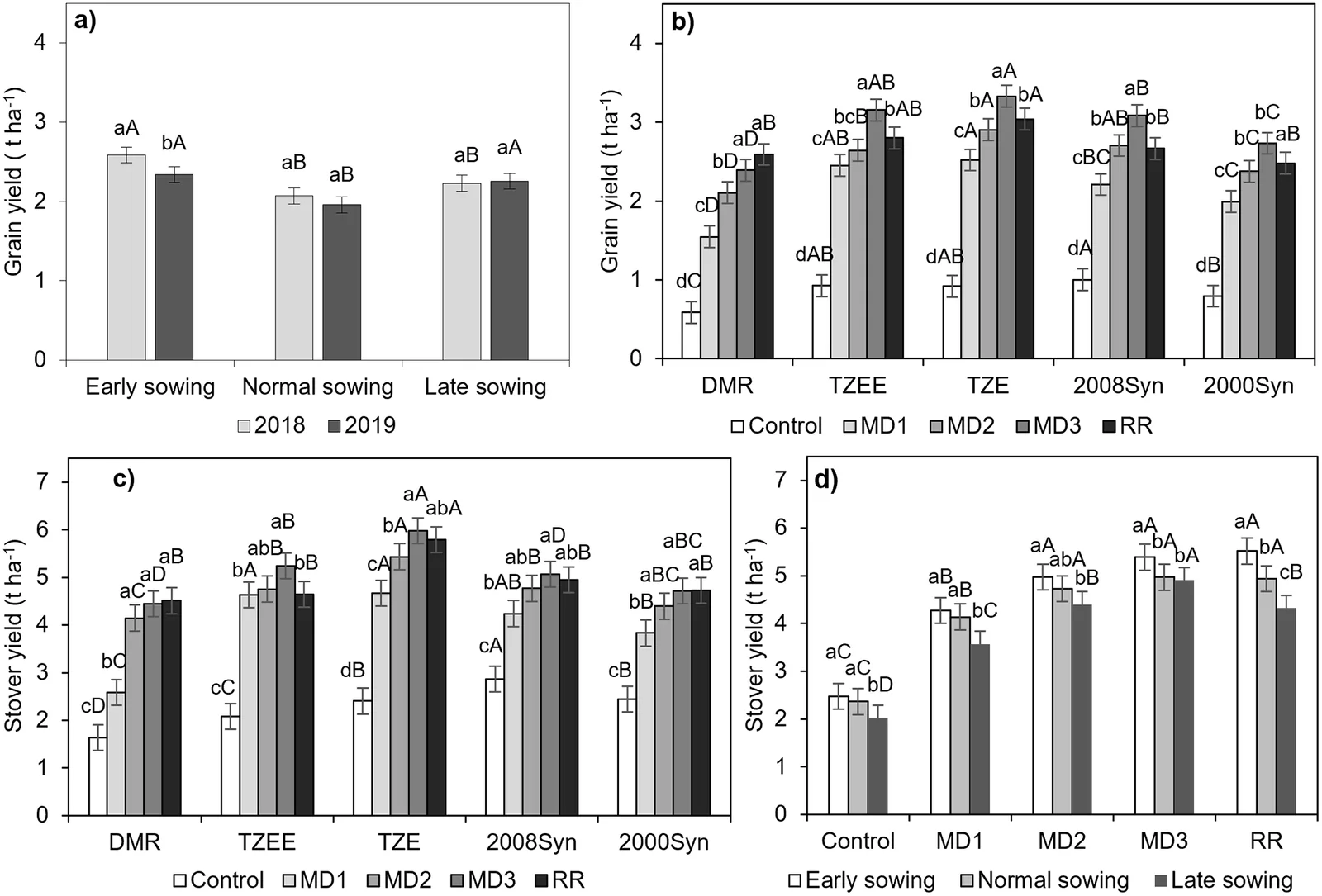
Grain yield (a) and stover yield (b, c) as affected by the interaction between variety and fertilizer (a and b) and, sowing date and fertilizer (c) maize. GY: grain yield, SY: stover yield. Control: unfertilized, MD1: 25 kg N + 4 kg P ha^-1^, MD2: 25 kg N + 8 kg P ha^-1^, MD3: 35 kg N + 8 kg P ha^-1^, RR: 76 kg N + 13 kg P ha^-1^. Error bars represent the standard error. Letters “abc” indicate mean separation among the grouped bars, while letters “ABC” indicates mean separation among factor represented in the legend (a > b > c, A > B > C).

### Effect of sowing date, drought tolerant varieties and fertilizer microdosing on agronomic efficiency and partial factor productivity

Agronomic efficiency of nitrogen (AE-N) and phosphorus (AE-P) were both higher under early (9.8% and 10.0%), and late sowing (3.7% and 2.9%) compared to normal sowing, respectively (Table 3). Both AE-N and AE-P increased by 7−36% for TZEE, TZE, 2008Syn, and 2000Syn compared with DMR (Table 3). AE-N was 31% and 15% higher under MD2 and MD3, compared to MD1, respectively; but 64% lower under RR. AE-P was 64%, 19% and 32% lower under MD2, MD3 and RR, compared to MD2, respectively. Both AE-N and AE-P varied significantly with the variety × fertilizer interaction (*P* < 0.001; Fig 7a and 7b). AE-N peaked under MD2 for DMR (60.8 kg kg^-1^), TZE (79.4 kg kg^-1^), 2008Syn (68.1 kg kg^-1^), and 2000Syn (63.4 kg kg^-1^), while for TZEE, AE-N did not differ significantly among MD1, MD2, and MD3. AE-P was highest under MD1 for TZEE (381.4 kg kg^-1^), TZE (403.2 kg kg^-1^), 2008Syn (301.8 kg kg^-1^), and 2000Syn (299.6 kg kg^-1^), with no significant difference between MD1 and MD3 for DMR.

**Fig 7:**
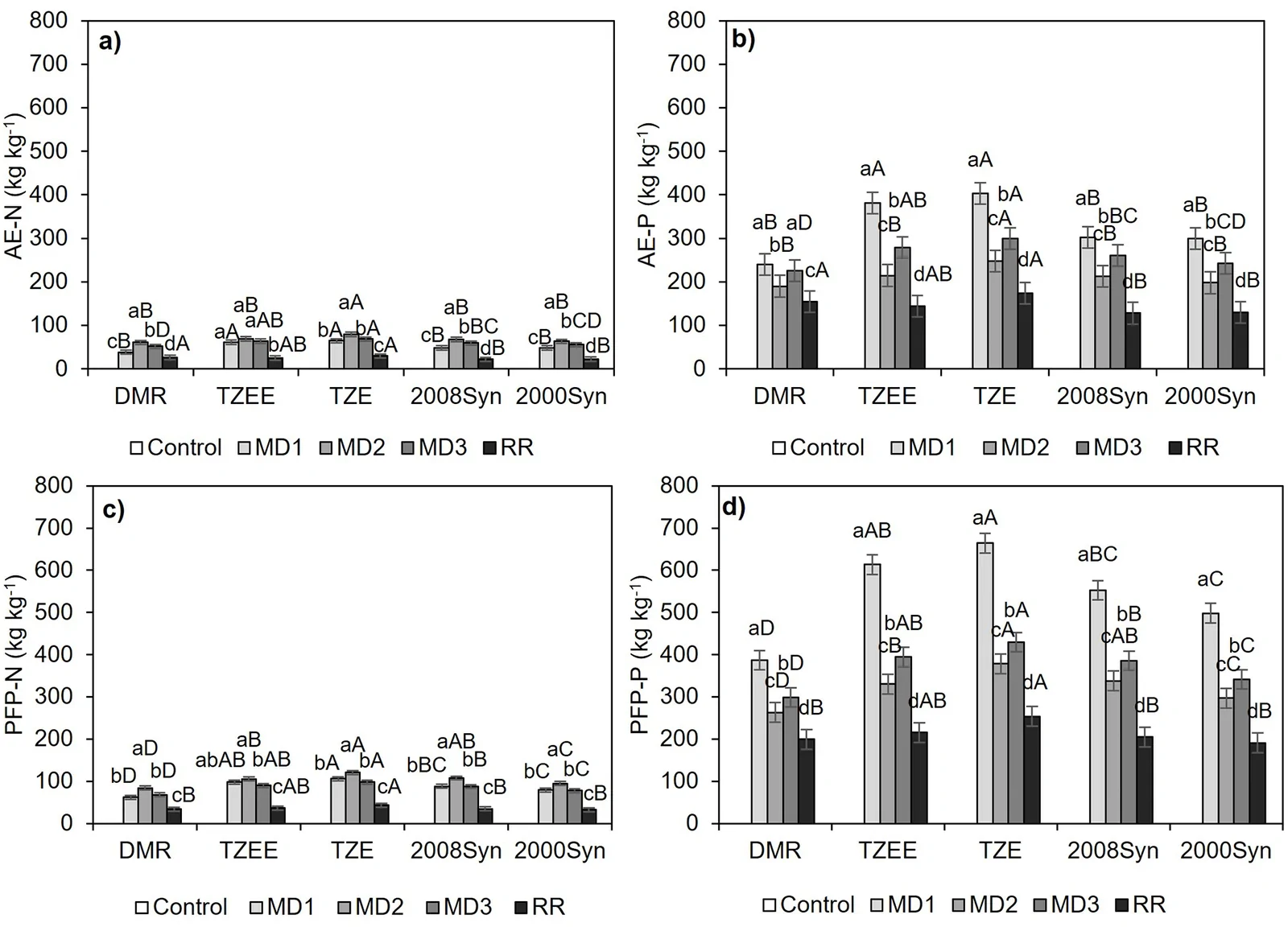
AE-N (a), AE-P (b), PFP-N (c) and PFP-P (d) as affected by the interaction between maize variety and fertilizer. AE: agronomic efficiency, PFP: partial factor productivity. Control: unfertilized, MD1: 25 kg N + 4 kg P ha^-1^, MD2: 25 kg N + 8 kg P ha^-1^, MD3: 35 kg N + 8 kg P ha^-1^, RR: 76 kg N + 13 kg P ha^-1^. Error bars represent the standard error. Letters “abc” indicate mean separation among the grouped bars, while letters “ABC” indicates mean separation among factor represented in the legend (a > b > c, A > B > C).

Partial factor productivity for nitrogen (PFP-N) and phosphorus (PFP-P) showed similar trends. PFP-N and PFP-P were 9.5% and 9.6% higher under early sowing than normal sowing, respectively, but both were 7.6% and 8.3% lower under late sowing (Table 3). PFP-N and PFP-P increased by 15−43% for TZEE, TZE, 2008Syn, and 2000Syn, compared to DMR. PFP-N was highest under MD1 (85.8 kg kg^-1^) and MD3 (83.9 kg kg^-1^) and lowest under RR (35.8 kg kg^-1^). Similarly, PFP-P was highest under MD1 (536.6 kg kg^-1^), followed by MD3 (367.5 kg kg^-1^), and lowest under RR (208.9 kg kg^-1^). Both indices differed significantly with the variety × fertilizer interaction (*P* < 0.001; Fig 7c and 7d) and followed the same overall trends as AE-N and AE-P, respectively.

### Redundancy analysis of sowing date, fertilizer microdosing, growth, yield and nutrient productivities of different maize varieties

Fertilizer microdosing and sowing date significantly influenced the growth, yield, and nutrient productivities of DMR maize (Fig 8). Growth and yield parameters, including TDM, LAI, GY, TGW, and SY clustered in the lower right quadrant of the ordination plot, indicating positive associations with both moderate (MD1, MD2) and higher fertilizer rates (MD3, RR), as well as with normal and early sowing. In contrast, nutrient productivity indicators (AEN, AEP, PFPN, PFPP) were more closely associated with lower fertilizer rates (MD1, MD2), as shown by their position in the upper right quadrant, while higher fertilizer rate (RR) had less favorable effects on nutrient efficiency.

**Fig 8:**
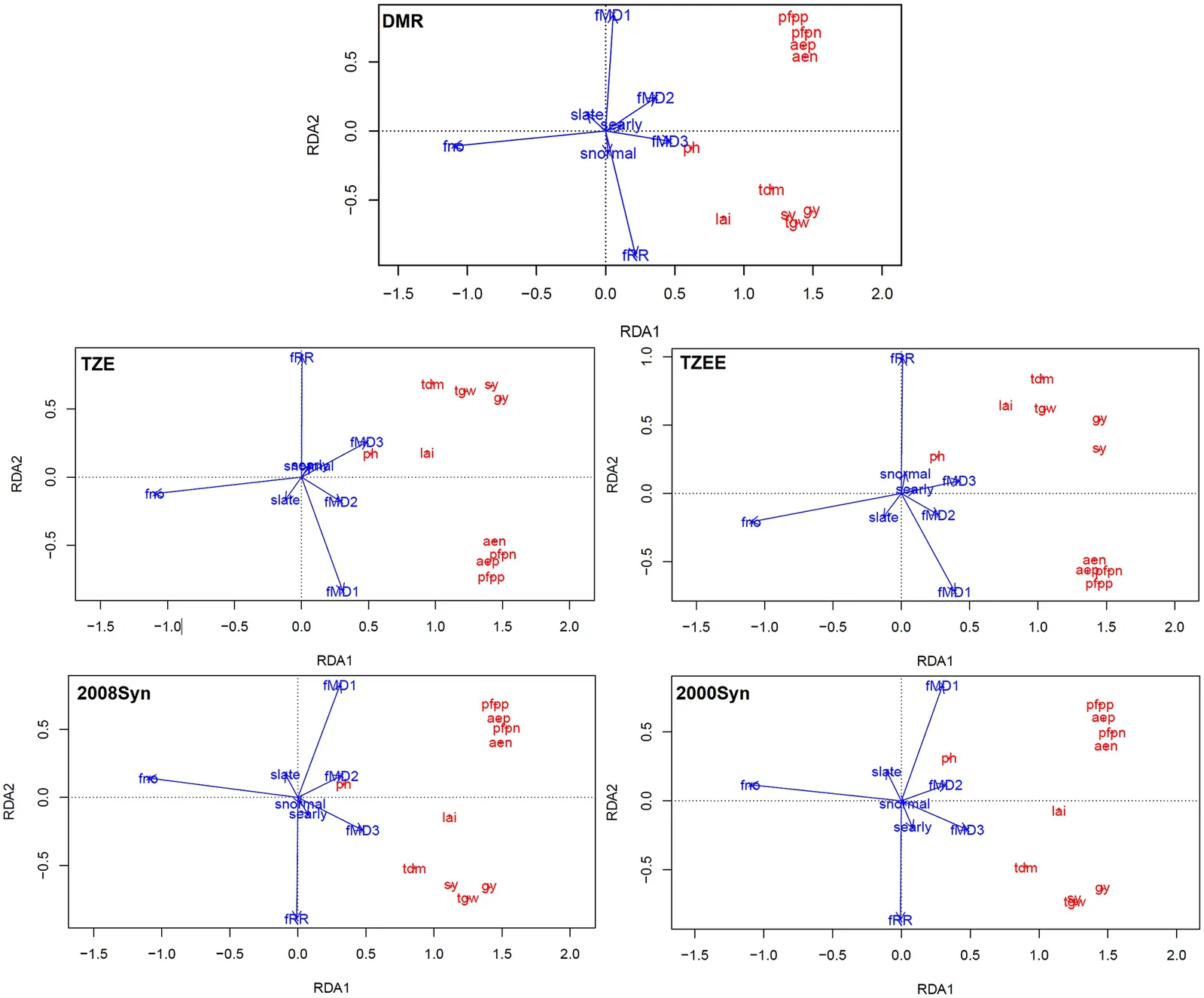
Redundancy analysis of maize management, growth, yield and nutrient productivities under drought-tolerant maize varieties. The RDA explained 73.9, 70.8, 70.5, 71.6 and 73.7% of the total variation in DMR, TZE, TZEE, 2008Syn and 2000Syn, respectively. The first two axes accounted for a cumulative proportion ranging from 62.3 - 65.5% of the variation.

Drought-tolerant varieties TZE and TZEE exhibited similar response patterns to fertilizer microdosing and sowing dates (Fig 8). For both, higher fertilizer rates (MD3 and RR) combined with early or normal sowing were positively correlated with growth and yield traits (TDM, LAI, GY, TGW, SY), whereas nutrient productivities (AEN, AEP, PFPN, PFPP) were more associated with lower fertilizer rates (MD1 and MD2) under early or normal sowing conditions. Control plots without fertilizer and those under late sowing showed negative associations with both yield and nutrient efficiencies. Comparable trends were observed for the drought-tolerant hybrids 2008Syn and 2000Syn (Fig 8).

## Discussion

### Early sowing and fertilizer microdosing enhance maize growth and yield

Maize vegetative growth responded strongly to sowing date and fertilizer application, with early sowing and higher fertilizer rates consistently improving both parameters. The advantage of early sowing likely resulted partly from a more favourable distribution of rainfall across critical developmental stages. In particular, cumulative rainfall from tasselling through milking was 394.1 mm under early sowing compared with 314.6 and 151.6 mm under normal and late sowing, respectively, in 2018, and 154.4 mm compared with 101.1 and 131.7 mm, respectively, in 2019. Therefore, early-sown maize received approximately 25% and 53% more rainfall during this reproductive period than normal-sown maize in 2018 and 2019, respectively. Greater water availability during tasselling, silking and early grain filling may have favoured pollination, kernel establishment and assimilate supply to developing grains [48,49]. Conversely, the lower seasonal rainfall received by late-sown crops, particularly in 2018, likely increased the risk of moisture limitation during reproductive development [50]. The higher grain and biomass yields under MD3, particularly for the drought-tolerant varieties, could be attributed to improved nutrient use and a greater capacity to sustain reproductive growth under variable moisture conditions. Localized N and P placement concentrates nutrients near the root zone, thereby stimulating lateral root development, nutrient absorption and dry-matter accumulation [51,52]. Early sowing could have extended grain filling duration and improved harvest index [53,54], while drought-tolerant varieties efficiently remobilized nitrogen during grain filling and maintained photosynthetic activity under low-input conditions [55]. The combination of early sowing and fertilizer microdosing maximized both vegetative growth and biomass productivity. These results align with Djaman et al. [56], while other studies reported negligible or adverse effects of altered sowing times due to unfavorable moisture or temperature [57,58].

### Sowing date modulates fertilizer use efficiency through crop-nutrient synchronization

Nutrient use efficiencies, as indicated by AE and PFP, were consistently higher under early and normal sowing, compared to late sowing. This indicates that early planting favors improved resource utilization. Early sowing ensures better synchronization between nutrient availability and crop demand, particularly during the early vegetative and reproductive stages [59]. This pattern is consistent with Sarker et al. [60], who reported in sunflower that agronomic N efficiency was 18.6 and 15.9% higher under early and intermediate sowing compared to late sowing. The redundancy analysis further revealed that while higher fertilizer rates substantially increased biomass and yield, they reduced nutrient use efficiency due to excess nutrient availability. Conversely, moderate fertilizer microdosing rates (MD1 and MD2) provided a balanced response, sustaining strong growth and yield while enhancing nutrient utilization efficiency. These moderate rates likely prevent nutrient losses through leaching and maintain a steady nutrient supply throughout crop development [61]. Although higher fertilizer applications can boost yield, they tend to lower efficiency as excess nutrients remain unutilized [62,63].

### Drought-tolerant varieties exhibit superior adaptability and optimize yield

DMR achieved highest yields under the full recommended rate, while all drought-tolerant varieties performed best under MD3. This means that drought-tolerant varieties achieved equal or higher yields than DMR with 54% less nitrogen and 38% less phosphorus input. This superior nutrient use efficiency and adaptability to low-input conditions indicates that drought-tolerant genotypes have been bred to optimize resource utilization under low-input conditions [64,65]. Moderate microdosing rates (MD1 and MD2) balanced yield gains with nutrient efficiency, preventing excess nutrient availability while maintaining steady supply throughout crop development [55,66]. The fertilizer × sowing date interaction for stover yield suggests that compressed growing seasons under delayed planting may benefit from concentrated nutrient supply near the root zone through microdosing, enhancing uptake efficiency during shortened vegetative phases [67,68]. Late sowing reduced grain yield, while drought-tolerant varieties produced higher average yields than DMR. This consistent superiority indicates broad genetic adaptation to varying environmental conditions [69,70]. These findings align with previous studies demonstrating that drought-tolerant varieties outperform conventional ones under multiple stresses, including water, nutrient, and heat stress [71,72]. Early planting remains optimal for maximizing absolute yields [62], but the combination of drought-tolerant varieties with fertilizer microdosing could provide reliable productivity gains across variable planting windows.

### Implications and recommendations

These findings have important implications for fertilizer recommendation systems, which should differentiate between variety types rather than applying general recommendations. Farmers should prioritize early sowing whenever rainfall onset and field accessibility permit. The use of drought-tolerant varieties should also be promoted, in combination with the MD3 microdosing rate, which produced approximately 12% higher grain yield than the full recommended rate while requiring 54% less nitrogen and 38% less phosphorus. When timely sowing is not possible because of delayed or erratic rainfall onset, MD3 may still improve average grain yield relative to lower fertilizer rates or the unfertilized control. For farmers with more limited access to mineral fertilizer, MD2 offers a lower-input alternative, as it maintained approximately 93% of the grain yield achieved with the full recommended rate while using 67% less nitrogen and 38% less phosphorus. Few limitations should be considered when interpreting these results. This study was conducted on a single-site, thereby limiting extrapolation across diverse agroecological zones. Future research should conduct multi-location trials to validate these management strategies under varying environmental conditions and perform economic analyses to guide farmer adoption decisions. Furthermore, on-farm participatory validation studies would help identify context-specific barriers to the adoption of these management strategies.

## Conclusion

The study highlights the role of sowing date management, maize variety selection, and fertilizer application in sustaining maize productivity under unpredictable rainfed conditions. Early sowing increased grain yield by 9.9% compared to normal sowing, whereas late sowing reduced yield by 10.2%. Drought-tolerant varieties consistently yielded more than the drought-sensitive DMR-ESR-W variety, with TZE-Y POP STR and TZEE-W POP STR QPM showing the greatest average yield advantages of about 38% and 30%, respectively. Across drought-tolerant varieties, the microdosing rate of 35 kg N plus 8 kg P ha^-1^ produced approximately 12% higher grain yield than the full recommended rate of 76 kg N plus 13 kg P ha^-1^ (RR) while requiring 54% less nitrogen and 38% less phosphorus. The highest average grain yield was 3.51 t ha^-1^ with TZE-Y POP STR combined with 35 kg N plus 8 kg P ha^-1^ and early sowing, followed by 3.29 t ha^-1^ under normal sowing. Therefore, drought-tolerant maize varieties combined with 35 kg N plus 8 kg P ha^-1^ across each sowing date appears to be a promising lower-input management option in the northern Benin. Under the conditions of this study, these results highlight the potential of drought-tolerant varieties and targeted fertilizer management to reduce production risks, improve resource use efficiency, and enhance agricultural sustainability and resilience in rainfed systems facing climate variability. Further multi-location and multi-year field validation, combined with economic assessment under farmer-managed conditions, is needed to determine the robustness, profitability and wider applicability of this management option.
